# Lattice parameter accommodation between GaAs(111) nanowires and Si(111) substrate after growth via Au-assisted molecular beam epitaxy

**DOI:** 10.1186/1556-276X-7-109

**Published:** 2012-02-08

**Authors:** Anton Davydok, Steffen Breuer, Andreas Biermanns, Lutz Geelhaar, Ullrich Pietsch

**Affiliations:** 1 Festkörperphysik, Universität Siegen, Walter-Flex-Str. 3, Siegen, 57072, Germany; 2 Paul-Drude-Institut für Festkörperelektronik, Hausvogteiplatz 5-7, Berlin, 10117, Germany

**Keywords:** nanowires, MBE growth, X-ray diffraction

## Abstract

Using out-of-plane and in-plane X-ray diffraction techniques, we have investigated the structure at the interface between GaAs nanowires [NWs] grown by Au-assisted molecular beam epitaxy and the underlying Si(111) substrate. Comparing the diffraction pattern measured at samples grown for 5, 60, and 1,800 s, we find a plastic strain release of about 75% close to the NW-to-substrate interface even at the initial state of growth, probably caused by the formation of a dislocation network at the Si-to-GaAs interface. In detail, we deduce that during the initial stage, zinc-blende structure GaAs islands grow with a gradually increasing lattice parameter over a transition region of several 10 nm in the growth direction. In contrast, accommodation of the in-plane lattice parameter takes place within a thickness of about 10 nm. As a consequence, the ratio between out-of-plane and in-plane lattice parameters is smaller than the unity in the initial state of growth. Finally the wurtzite-type NWs grow on top of the islands and are free of strain.

## Introduction

Semiconductor nanowires [NWs] are of particular interest due to the ability to synthesize single-crystalline one-dimensional [1-D] epitaxial structures and heterostructures in the nanometer range. For the integration into high-performance III-V NW-based devices, such as light-emitting devices [1], high-mobility and high-frequency devices [2,3], and single-electron devices [4], on Si substrates, it is important to control NW morphology, dimension, uniformity, and electric and optical properties [5]. One route of NW growth is the vapor-liquid-solid [VLS] mode realized by metal-organic vapor phase epitaxy [6] or molecular beam epitaxy [MBE] by a solution from a molten eutectic alloy formed by a metallic seed. It was found that nearly any A^III^B^V ^semiconductor material can be grown as NWs onto another A^III^B^V ^or group IV (111) substrate independent from the lattice mismatch [7]. Independent from the mismatch, NWs are grown with high crystalline perfection except for the inclusion of stacking faults.

There is no systematic study of the lattice parameter accommodation at the NW/substrate interface which might be strongly linked to the growth process. For gold-assisted NW growth, several authors reported on alloy formation between the seed and the precursor material at the substrate/NW interface [8,9]. A growth scenario via islands towards NWs has been proposed for gold-assisted MBE growth of GaAs NWs onto Si(111) measuring samples of different growth times [10]. By scanning electron microscopy [SEM] inspection, wormlike GaAs aggregates were found at the early stage of growth, which were increasing in size to form islands before the real GaAs NW growth takes place. However, although reflection high-energy electron diffraction and high-resolution transmission electron microscopy [HRTEM] could identify the evolution of the crystal structure to be zinc blende [ZB] in traces and islands but wurtzite [WZ] in NWs, the strain release between the substrate and NWs could not be quantified. This missing information motivated additional high-resolution X-ray diffraction [HRXRD] measurements at similar GaAs NWs as inspected in the work of Breuer et al. [10] using synchrotron radiation. For these measurements, we selected samples with extreme differences in growth time ranging from 5 s to 30 min and used high-resolution out-of-plane and grazing incidence in-plane X-ray diffraction [GIIXD] techniques to evaluate the strain status in both directions. In this paper, we will show that NWs grow nearly free of strain on top of GaAs islands which accommodate the complete lattice mismatch between GaAs and the Si substrate. The strain release is partially plastic close to the NW-to-substrate interface followed by the gradual increase of lattice parameters towards the top of the islands.

## Experimental details

The investigated GaAs NWs were grown by MBE at the Paul-Drude-Institut (Berlin, Germany) using the Au-assisted VLS mechanism. As substrate, we used *n*-type Si(111) ± 0.5° substrates whose native oxide was removed using different chemical agents like Ga (*in situ*) or HF and NH_4_F (*ex situ*). Further details of the growth procedure are given in the work of Breuer et al. [10].

For our investigation, we selected two series of samples. The substrate of the first three samples (A1 to C1) were etched by NH_4_F + HF (sample A1: growth time 5 s; sample B1: growth time 60 s) and by HF (sample C1: growth time 30 min).

For comparison, a second series of samples (a2 to c2) with different surface preparations but otherwise identical growth conditions compared to samples A1 to C1 have been investigated. Whereas samples a2 and b2 were cleaned *in situ *by depositing Ga and desorbing the resulting oxide, sample c2 was prepared using NH_4_F instead of HF as done for C1. The X-ray measurements of samples A1 and B1 were performed about 4 weeks after growth, whereas the other samples were measured 14 months after growth.

**Figure 1:**
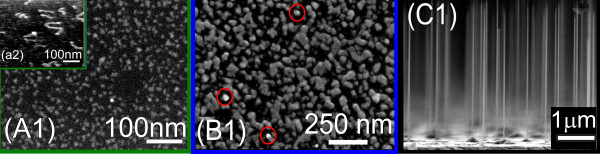
**SEM images of GaAs NWs**. These NWs were grown for 5 s (samples A1 and a2), 60 s (sample B1), and 1,800 s (sample C1). During the initial stage of growth, GaAs traces are formed (sample a2), as seen in the inset (A1), evolving into island type structures and finally nanowires. Images of samples A1 and B1 represent top views, whereas the sample C1 image is shown in cross-sectional view in order to visualize the vertical nanowires.

All samples have been pre-characterized by SEM. Figure 1 demonstrates the results of SEM inspections of samples A1, a2 (inset), B1, and C1,respectively. Figure 1 (A1) exhibits few trace-like patches with lengths of 50 to 100 nm that spread over the surface. The number of such traces is large, and most of them show a bright spot at one end, marking the position of the Au droplet. The inset in Figure 1 shows a magnified view of the surface of sample a2. In this case, the surface coverage is reduced, making the elongated traces more clearly visible. For longer growth time, the GaAs traces are enlarged and reach a diameter of about 50 nm, whereas the total surface coverage reaches the order of 50%. An individual trace may grow laterally via the VLS mode through the Au droplet as long as it becomes not encompassed by a neighboring trace. When lateral growth stops, the traces act as the base for vertical NW growth. This initial state of NW growth is displayed in Figure 1 (B1) by bright spots marked by red circles. Finally, Figure 1 (C1) shows NWs of about 3 μm in height, and an average diameter of 25 nm grown onto islands are found in Figure 1 (B1). However, the number density of islands without NWs exceeds that of those hosting NWs. The NW density is about 1.3 μm^-2^. The NW density reached in sample c2 is about 10 times smaller (not shown). Qualitatively similar features are seen on SEM images of samples cleaned with Ga as shown in the work of Breuer et al. [10].

**Figure 2:**
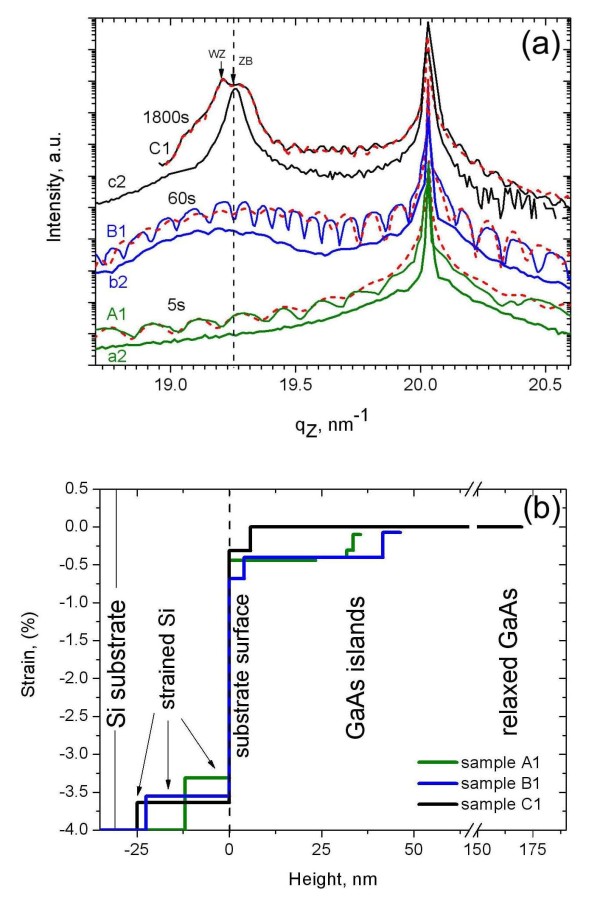
**HRXRD curves and lattice parameters**. (**a**) HRXRD curves of all samples; the dotted lines are the results of simulations. (**b**) Lattice parameters as a function of height above the Si surface were obtained by the simulations of the curves shown in (a). Values are shown as the lattice parameter difference with respect to the unstrained GaAs lattice. A thickness up to 25 nm below the Si surface is affected during the GaAs growth. The GaAs lattice shows a gradual increase in lattice parameter.

Figure 2a shows the radial HRXRD scans through the Si(111) Bragg peak for the six samples. All samples display the Si(111) peak at *q*_z _= 20.03 nm^-1^. After 5 s of growth (sample A1), the intensity distribution already becomes asymmetric with respect to the substrate peak, i.e., the intensity of the tail at the lower *q*_z _side is higher than that at the larger *q*_z _side. As a peculiarity of the sample series (A1 to C1), moderate oscillations are seen, indicating the presence of a near surface layer with different scattering contrasts (see 'Discussion' section). With increasing growth time, a peak develops close to the position of bulk GaAs(111) with *q*_zbulk(GaAs) _= 19.25 nm^-1 ^for samples B1 and C1. At the same time, the period of thickness oscillations for B1 becomes shorter compared with A1, corresponding to an increase of layer thickness. For C1, the GaAs peak can be decomposed into two sub-peaks with different lattice parameters for ZB and WZ (see 'Discussion' section). The fine structure of the measured HRXRD curves changes for measurements at different positions of the same sample and among different samples due to the different number density and size distribution of islands and NWs. As an example, we show the HRXRD curves of the second series (samples a2 to c2). In general, they reveal the same tendencies as seen for A1 to C1 except that the thickness oscillations do not occur and that only a single GaAs-related peak is found for sample c2. The latter difference between samples C1 and c2 is caused by the different number density of NWs. The separation of the two components seen for sample C1 in Figure 2a is highlighted by arrows. They are cantered at *q*_z _= 19.20 nm^-1 ^and *q*_z _= 19.28 nm^-1 ^and are attributed to the different out-of-plane lattice parameters of ZB (islands) and WZ (NWs) units, respectively [11]. The larger out-of-plane lattice parameter of WZ compared to ZB [12] leads to a separation of the Bragg peaks. The WZ peak disappears in c2 because the number density of NWs in this sample is by factor 10 smaller compared with C1 and does not contribute to the scattering signal. Whereas the ZB peak of sample c2 corresponds to the lattice parameter of bulk ZB GaAs, the ZB lattice parameter seen for C1 is slightly smaller probably due to the contribution of strain fields in the large number of islands on this sample.

**Figure 3:**
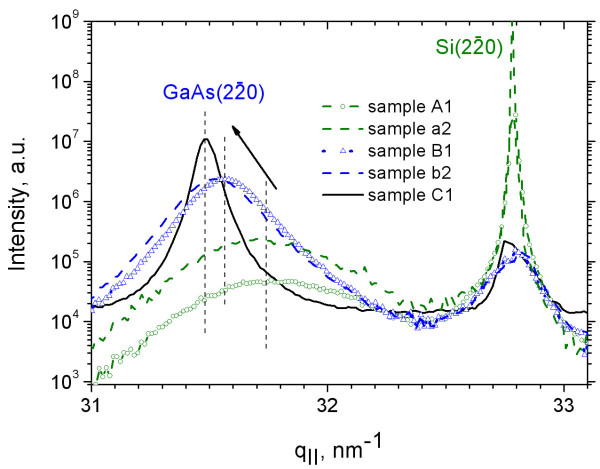
**Diffraction profiles**. Diffraction profiles through the in-plane (2-20) reflection of samples A1, B1, C1, a2, and b2 taken at *α*_∥ _= 0.15°. With increasing growth time, the islands grow and relax laterally towards the bulk lattice parameter reached for sample C1 (arrow).

The evolution of the in-plane lattice mismatch is measured at the ZB (2-20) reflections of Si and GaAs in GIIXD geometry, using an angle of incidence of the incoming X-ray beam with respect to the substrate close to the critical angle of the total external reflection (*α*_c _= 0.2° for Si at the used X-ray energy). The measured diffraction curves are shown in Figure 3. In contrast to the HRXRD experiments, the diffraction curves taken in GIIXD geometry do not differ so much among the different sample series and different probing areas, but they clearly show a dependence on growth time. The position of the bulk Si peak at *q*_∥ _= 32.72 nm^-1 ^was used as a reference for all samples. Compared with the curves of samples B1 and C1, the higher substrate intensity for sample A1 is explained by the lower coverage of the substrate by GaAs.

**Figure 4:**
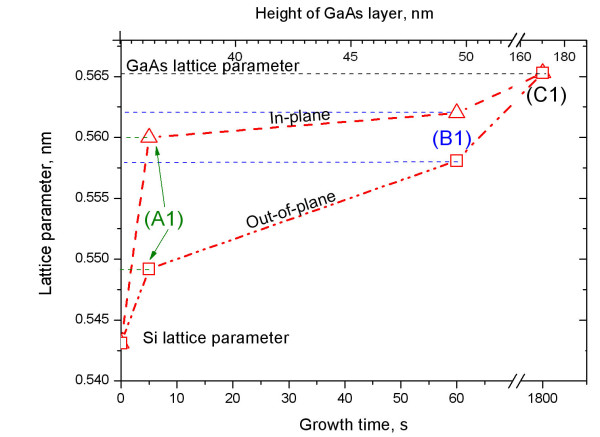
**Lattice parameters of the GaAs islands as a function of island-height/growth time**. The evolution of in-plane (triangles) and out-of-plane (squares) lattice parameters are shown. Along the in-plane direction, the lattice parameter increases faster during the initial stage of growth (sample A1). Dotted lines are guides to the eye.

For the sample with the lowest amount of GaAs (A1), the GaAs peak appears as a broad peak with full width at half maximum of Δ*q*_∥ _= 0.49 nm^-1 ^cantered at *q*_∥ _= 31.77 nm^-1 ^(see black dotted line in Figure 3), which is well isolated from the substrate peak. The same holds for sample a2. For further comparison, the measured peak position can be attributed to a cubic lattice parameter [*a*_∥GaAs_]. As shown in Figure 4, this deduced in-plane lattice parameter of *a*_∥GaAs _= 0.560 nm corresponds to a lattice mismatch of -1% with respect to bulk GaAs, i.e., a plastic strain release of 75% of the total lattice mismatch with respect to the substrate in the early phase of growth. With increasing growth time (samples B1 and b2), the GaAs peak becomes sharper and shifts towards the smaller *q*_∥ _= 31.57 nm^-1 ^(see arrow in Figure 3), i.e., to *a*_∥GaAs _= 0.562 nm. This peak can be decomposed into two: the peak measured at A1 (characterizing the GaAs islands) and the GaAs peak from sample C1 (characterizing both islands and NWs). The peak of sample C1 is cantered at *q*_∥ _= 31.49 nm^-1^, corresponding to the position of fully relaxed ZB GaAs with *a*_∥GaAs _= 0.565 nm. The respective peak width is reduced to Δ*q*_∥ _= 0.08 nm^-1^. Figure 4 summarizes the evolution of the in-plane lattice parameter as a function of growth time and correlates them with the height of the islands and lattice parameters deduced from the HRXRD curves in Figure 2. Sample c2 has not been measured by GIIXD.

The instantaneous jump of the lattice parameter straight after the beginning of growth is most likely caused by misfit dislocations at the island-to-substrate interface. Considering the grain size of about $\frac{2\pi }{\Delta q_{∥}}\approx 10\text{ nm}$ measured for samples A1 and a2, the mean distance between neighboring dislocations is estimated to be about 10 nm. Both curves taken for B1 and b2 show an asymmetry in peak shape with a larger width towards the larger *q*_∥_, indicating the appearance of an in-plane lattice parameter gradient from the initial state close to the substrate towards a bulk-like parameter on top of the GaAs islands. This elastic transition layer for the in-plane lattice parameter is in the order of about 10 nm.

## Methods

HRXRD measurements were performed at the ID01 beamline of ESRF, Grenoble, France. The incoming monochromatic X-ray beam with energy of 8 keV was collimated to a size of 100 × 100 μm^2 ^using a set of slits. To record the diffracted intensity, we used a 2-D pixel detector, MAXIPIX (ESRF, Grenoble, France), with a pixel size of 55 × 55 μm^2 ^[13].

Intensity profiles normal to the surface were measured in diffraction *θ*-2*θ *geometry (radial scans) with angular steps of Δ*θ *= 0.005°, subsequently changing the length of the scattering vector $q_{\text{z}}=\left(\frac{\text{4}\pi }{\lambda }\right)\text{sin}\left(\theta \right)$, where λ is the wavelength of the X-ray beam. Therefore, the measurement is sensitive to changes of the lattice parameter along the growth direction.

Complementarily, the in-plane lattice parameters were probed by grazing incidence diffraction at the P08 beamline of PETRA III (Hamburg, Germany) [14] at 8.94 keV in Bragg scattering geometry using incidence and exit angles of the X-ray beam with respect to the surface smaller than the critical angle of total external reflection in order to reduce the depth sensitivity to a few nanometers below the surface. Here a 1-D Mythen detector was used to resolve the diffracted intensity along the exit angle.

## Discussion

Our GIIXD data give evidence that during the initial state of growth, 75% of the lattice mismatch between GaAs and Si are already plastically released. The fast relaxation indicates the presence of misfit dislocations at the interface which have been observed by transmission electron microscopy [15]. The in-plane lattice parameters have been deduced from the (2-20) peak positions without curve simulation. This is justified by the large peak separation between the GaAs and Si substrate peaks and the thin transition layer.

In contrast to this, the out-of-plane lattice parameter varies over a GaAs equivalent layer thickness of several 10 nm. Its functional dependence is extracted from the HRXRD data shown in Figure 2a by means of a rocking curve simulation. The calculations have been performed in terms of the Tagaki-Taupin approach [16,17] using the software package RefSim, dividing the surface structure vertically into four layers with homogeneous density and gradually changing the lattice parameter from Si towards that of bulk GaAs. During simulation, no reasonable fit could be obtained by a single-layer model. The strain profiles normalized to the bulk value of GaAs as obtained by this simulation are shown in Figure 2b for the simulated curves shown in Figure 2a. The plot shows the evolution of lattice parameters as a function of height above the initial Si surface. Note that in this representation, the undisturbed Si substrate has a strain value of -4%.

As shown by the line profiles in Figure 2b, the simulations reveal that a part of the Si lattice below the initial surface is already affected by the growth, showing a layer with a slightly increased lattice parameter compared with that of the bulk Si. The thickness of this layer is gradually increasing with the growth time. The lattice expansion in this layer might be explained by Au inclusions due to enhanced Au diffusion at elevated growth temperatures into a layer containing a network of dislocations as discussed previously. The appearance of this deformed Si layer becomes visible because of the thickness oscillation seen for samples A1 to C1. The oscillation period decreases with the increasing growth time and measures an increasing thickness of this layer. Unfortunately, the detailed intensity distribution cannot be reproduced in a perfect manner for samples A1 and B1, but this discrepancy is of less importance for the further interpretation of the measured lattice parameters (see further details in the 'Discussion' section).

The absence of those oscillations on samples a2 to c2 can be explained by a laterally less homogeneous deformed Si layer. However, we find a good fit to the data considering the same deformed Si layers as found for samples A1 to C1.

On top of the deformed Si layer, the GaAs islands show an abrupt increase of lattice parameter, followed by a gradual increase towards the bulk lattice parameter. This is in general agreement with the findings from the GIIXD measurement.

We have to note that due to the phase problem of X-ray scattering, the proposed structure model is not unique. The results of the fitted model presented in Figure 1 were achieved, considering essential growth parameters like the amount of GaAs deposited in different stages of growth (planar growth rate 0.11 nm s^-1^), the size of gold droplets (approximately 10 nm), and the estimation of the surface roughness (approximately 5 to 10 nm during the first few seconds) from SEM and atomic force microscopy measurements.

The strained region within the GaAs islands has a thickness of about 10 nm for sample A1, 35 nm for sample B1, and 140 nm for sample C1 which is in good agreement with the cross-sectional HRTEM investigations [10]. The evaluated average thicknesses of the strained regions found for samples a2 and b2 are similar to those deduced for samples A1 and B1. This similarity indicates that the lattice relaxes during island and NW growths essentially independent from the surface treatment.

As already done for GIIXD data, the mean peak positions can be attributed to a cubic lattice parameter *a*∥_GaAs _within the islands. This lattice parameter is shown together with the GIIXD results in Figure 4. In samples A1 and a2, the vertical lattice parameter reaches *a*∥_GaAs _= 0.5492 nm at the top part of the islands corresponding to lattice compression of 2.8% with respect to bulk GaAs. For samples B1, b2, and C1, the lattice parameters on top of the islands are *a*∥_GaAs _= 0.5581 nm (compression 1.3%) for B1 and *a*∥_GaAs _= 0.5653 nm for b2. It means that the lattice parameter on top of the islands of sample C1 equals the value known for bulk GaAs as seen in Figure 2b. Comparing the results for all samples, both in-plane and out-of-plane lattice parameters are increasing from silicon towards the top of the islands. However, the increase is faster along the in-plane direction compared with the out-of-plane direction. After normalization by the undisturbed c/a ratio of 1.633 due to [111] growth direction, the ratios between out-of-plane and in-plane deformations can be expressed by *a*∥ and *a*∥, measured by HRXRD and GIIXD and collected in Figure 4. Due to the possibility of lattice expansion towards the island side planes, this ratio *a*∥_GaAs_/*a*∥_GaAs _is never larger than the unity as expected for a strained epitaxial layer with *a*_layer _>*a*_substrate_. In contrast, the ratio *a*∥_GaAs_/*a*∥_GaAs _is smaller than the unity in the initial stage of growth. It amounts to about 0.98, 0.99, and 1.0 for samples A1, B1, and C1, respectively, measured on top of the islands. In the second series, the ratio is 0.98 for a2, but it is already 1.0 for b2. Values for samples C1 and c2 prove again that NWs grow on completely relaxed GaAs islands.

## Conclusions

In summary, for the presented growth conditions, the complete lattice mismatch between GaAs and Si is released within the GaAs islands. About 75% of the mismatch between GaAs and silicon are released plastically, followed by an elastically decaying displacement field which is in the order of 10 nm in plane, but several 10 nm out of plane, resulting in a hexagonal distortion of the unit cell parameters with ratio *a*∥_GaAs_/*a*∥_GaAs _smaller than the unity but close to the island-to-substrate interface. The origin of plastic relaxation is probably the inclusion of a dislocation network. Subsequently, NW growth takes place on top of these islands which explains the absence of strain and their high crystal perfection.

## Competing interests

The authors declare that they have no competing interests.

## Authors' contributions

AD, AB, and UP carried out the X-ray investigation and data simulation. SB and LG carried out NW growth by MBE. All authors read and approved the final manuscript.
